# Supplementary figures and images for: Mechanistic and evolutionary insights into isoform-specific ‘supercharging’ in DCLK family kinases
Source: eLife. 2023 Oct 26;12:RP87958. doi: 10.7554/eLife.87958 (PMC10602587; doi:10.7554/eLife.87958)

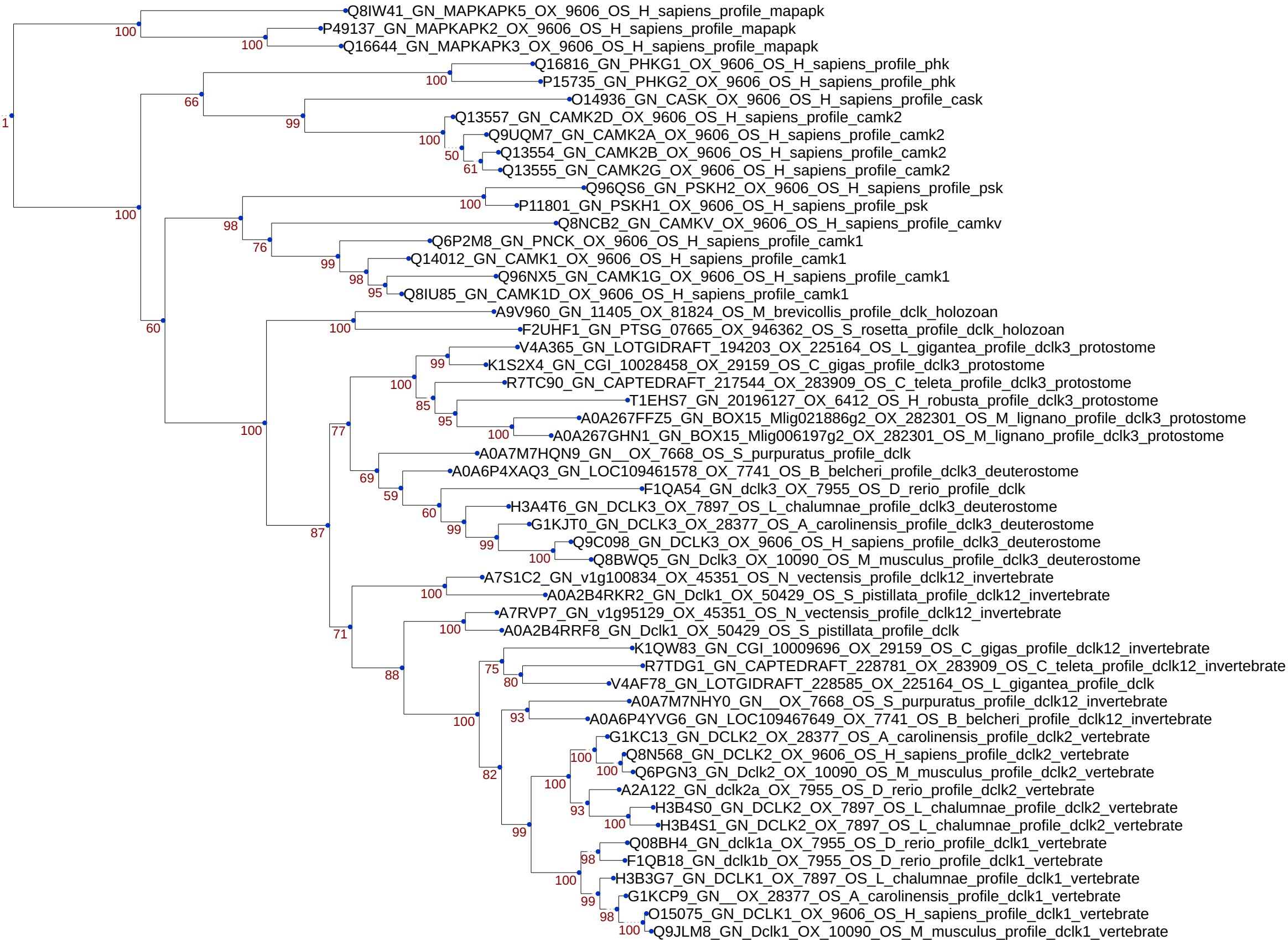

0.2

Supplement: Figure 2—source data 1. [file elife-87958-fig2-data1.pdf]

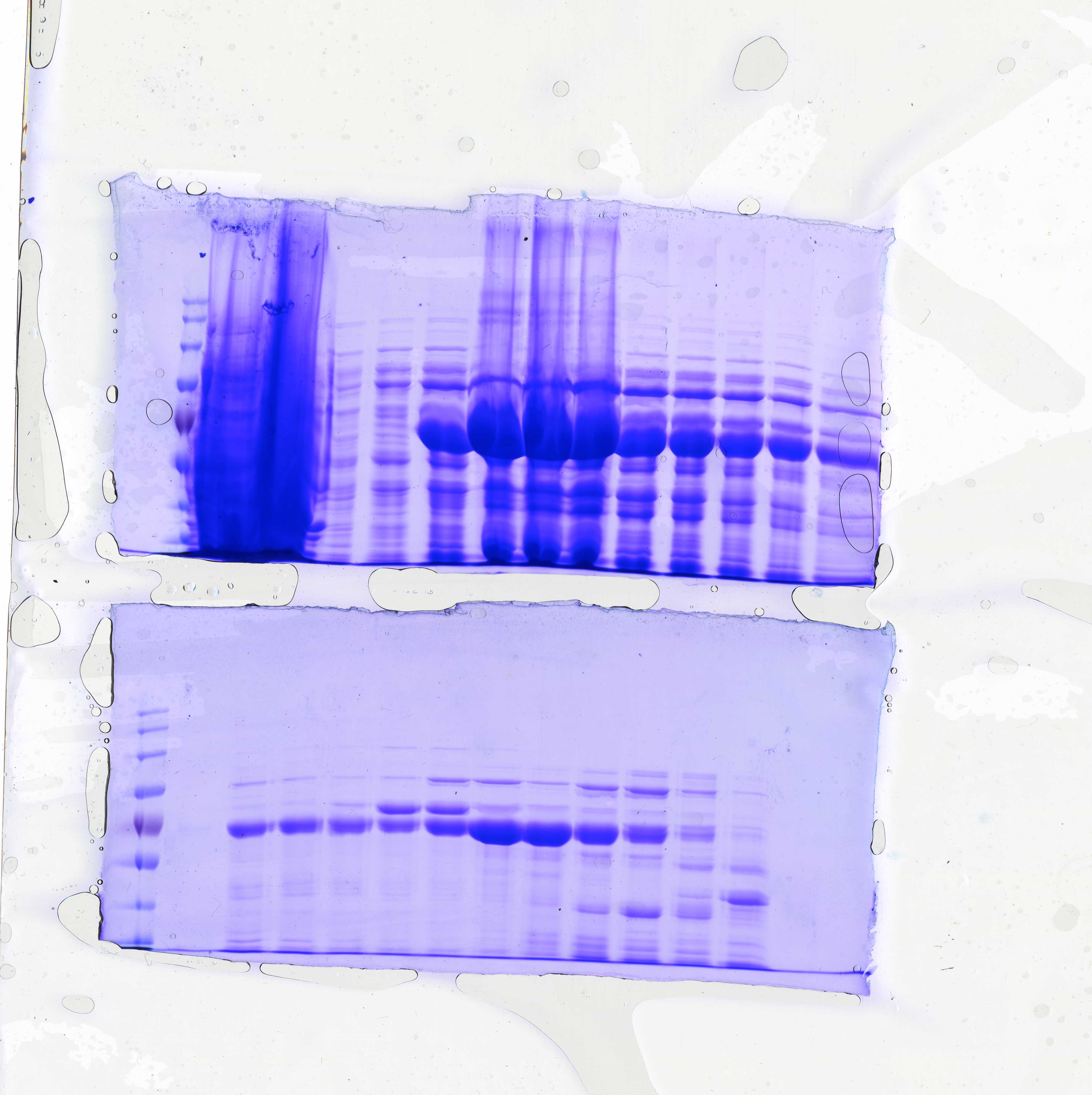

Supplement: Figure 3—source data 4. [file elife-87958-fig3-data4.zip › Figure3-SourceData4.jpg]

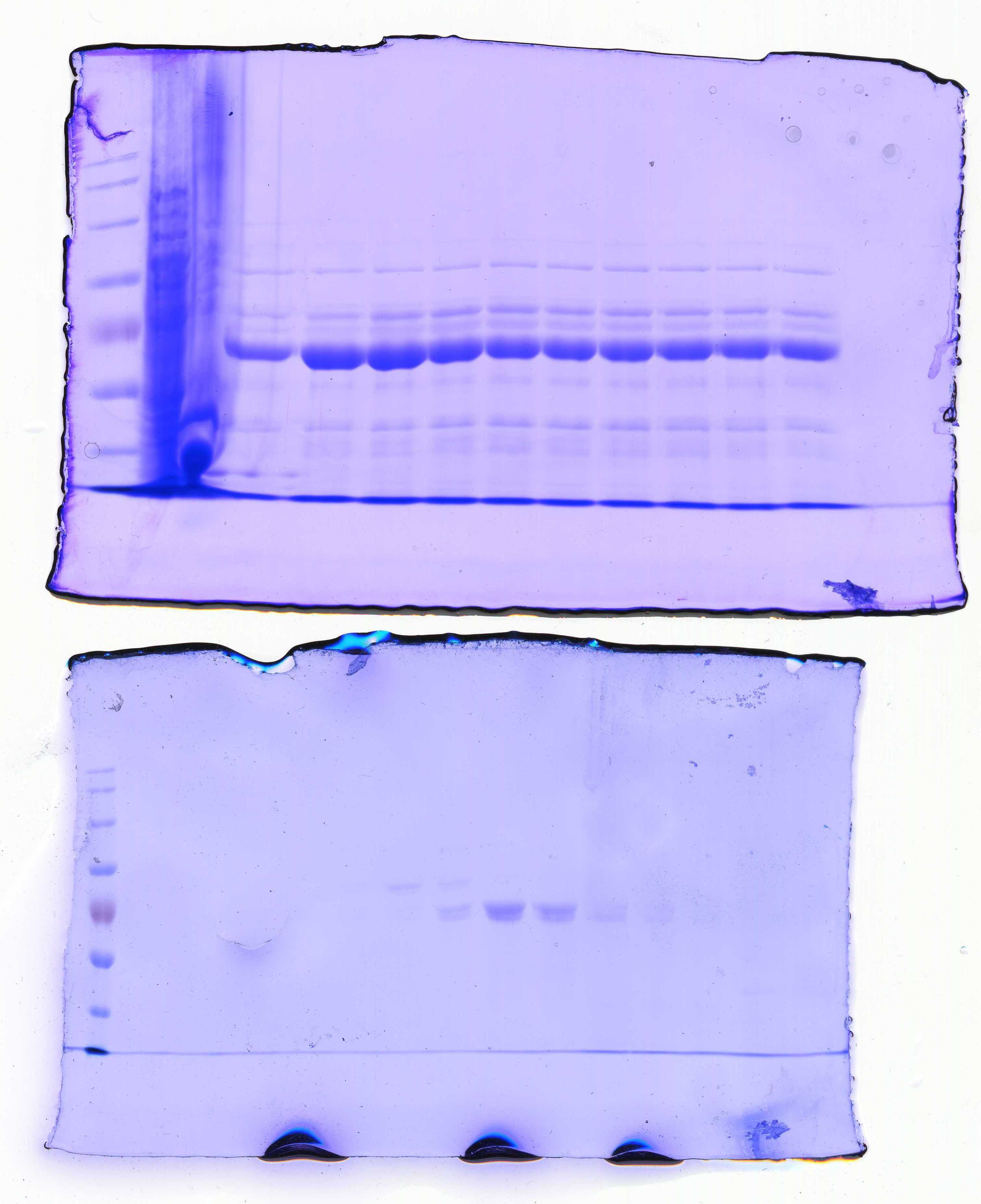

Supplement: Figure 3—source data 5. [file elife-87958-fig3-data5.zip › Figure3-SourceData5.jpg]

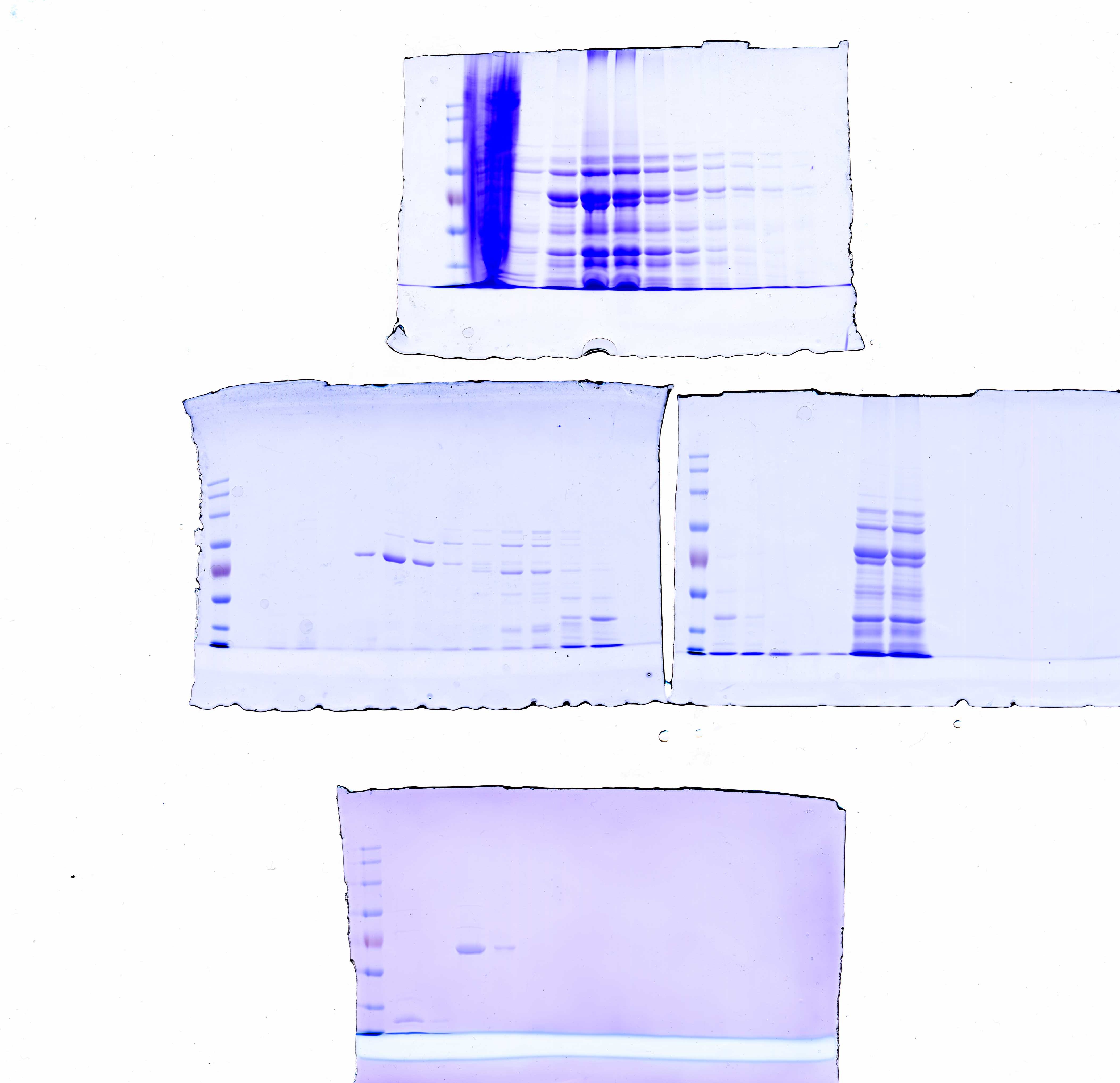

Supplement: Figure 3—source data 6. [file elife-87958-fig3-data6.zip › Figure3-SourceData6.jpg]

## Slide 1
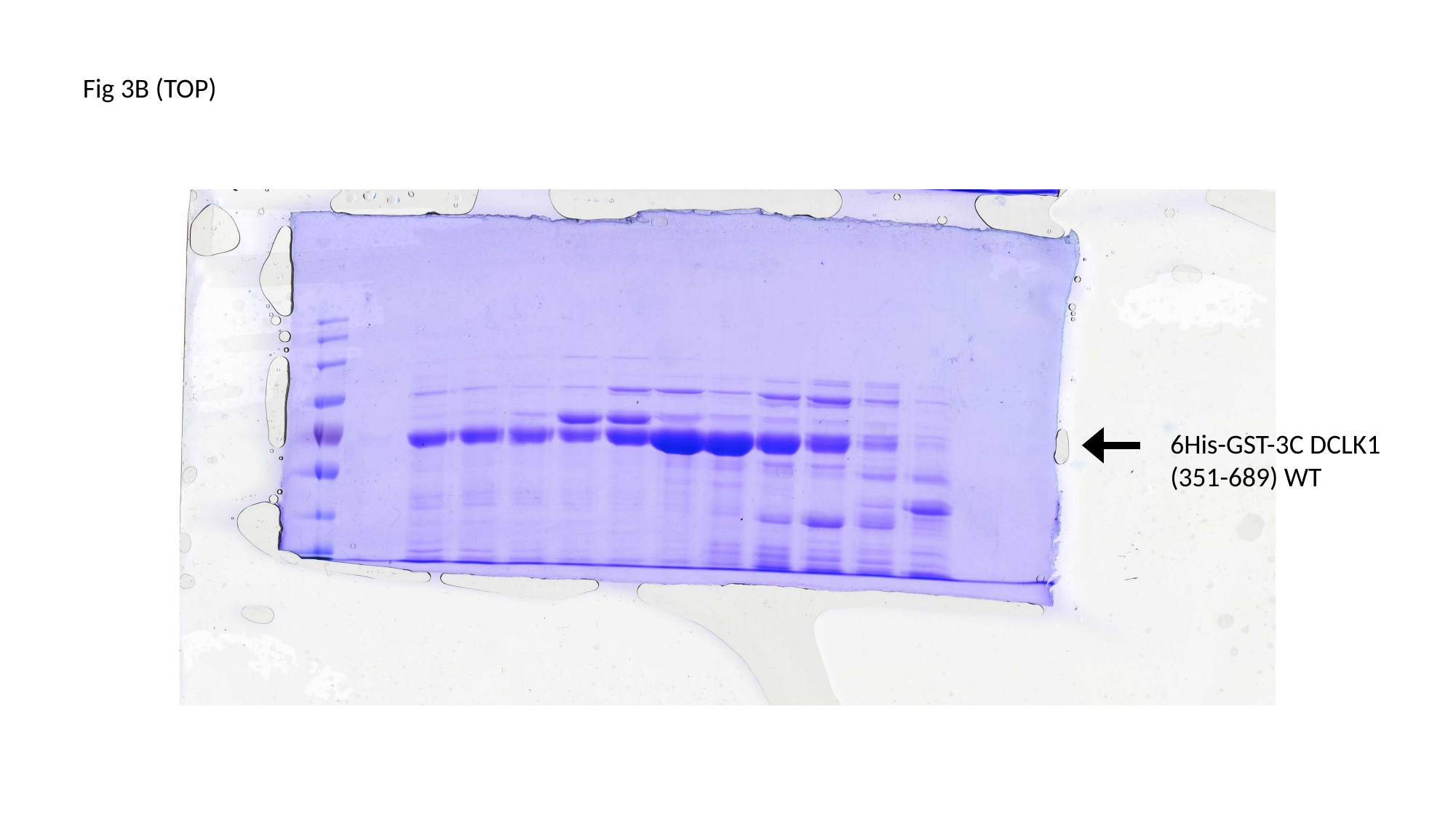

Fig 3B (TOP)
6His-GST-3C DCLK1
(351-689) WT

## Slide 2
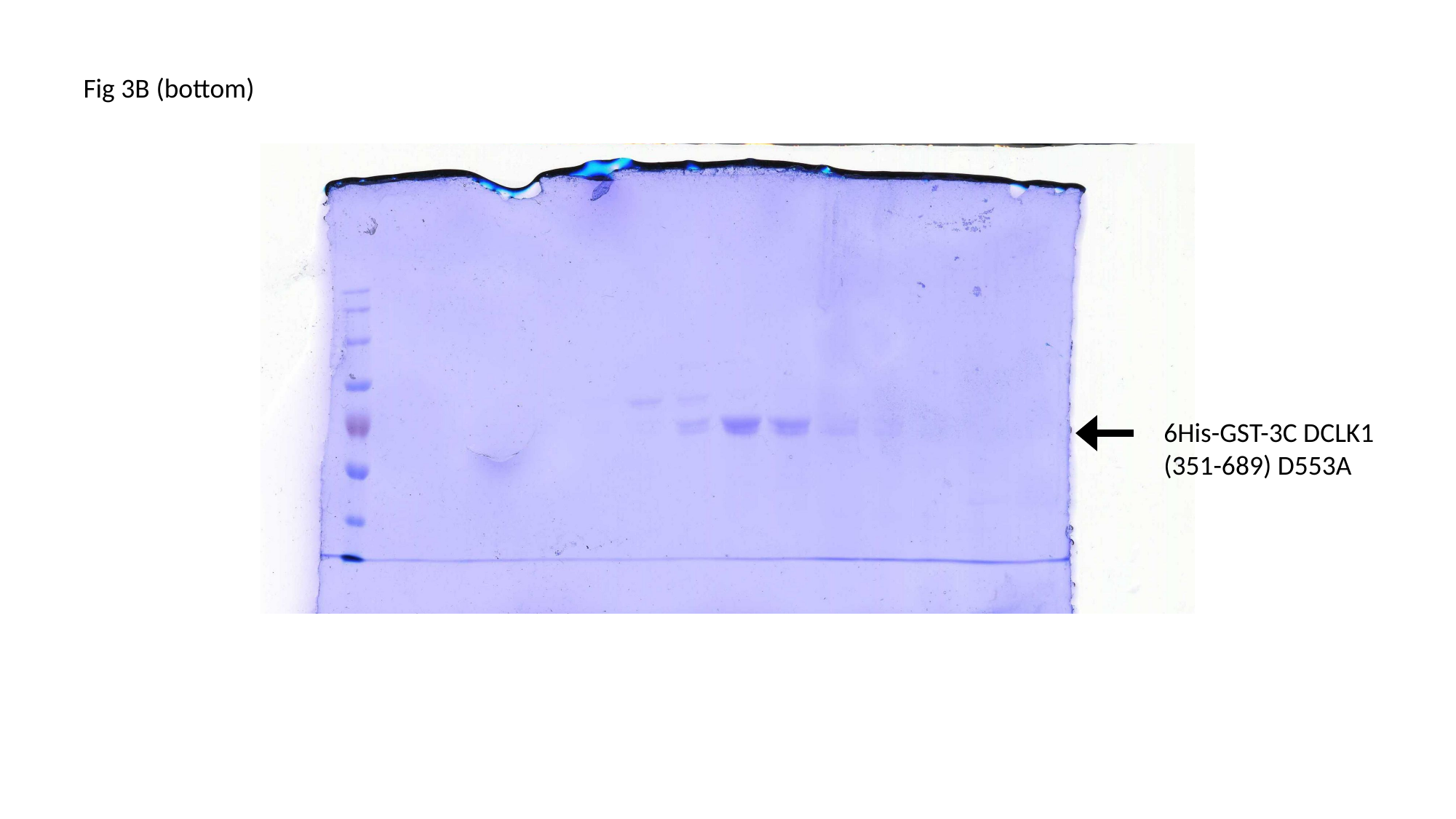

Fig 3B (bottom)
6His-GST-3C DCLK1
(351-689) D553A

## Slide 3
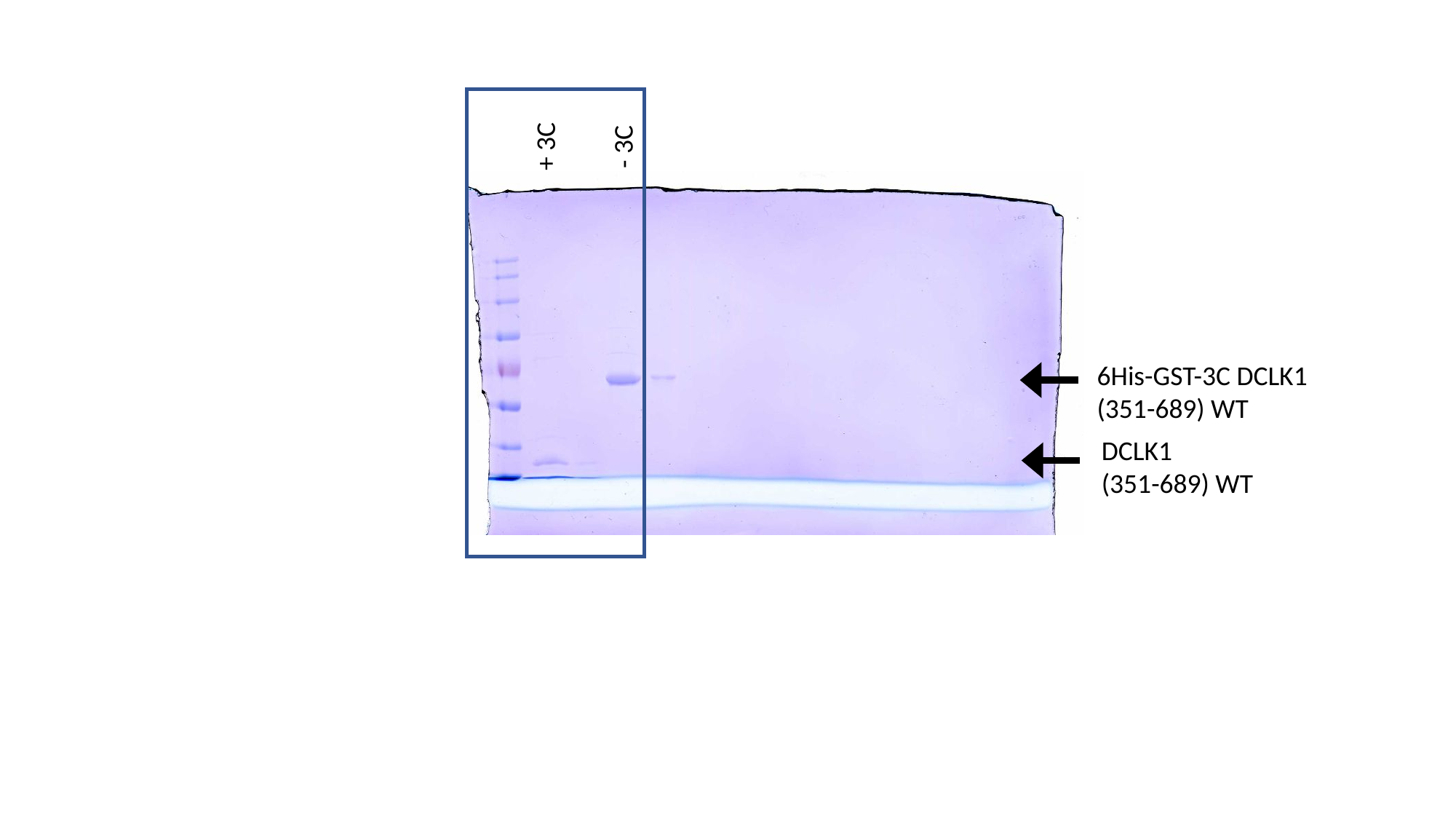

+ 3C
- 3C
6His-GST-3C DCLK1
(351-689) WT
DCLK1
(351-689) WT

Supplement: Figure 3—source data 7. [file elife-87958-fig3-data7.pptx]

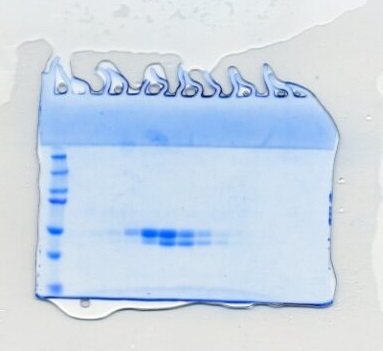

Supplement: Figure 3—figure supplement 1—source data 1. [file elife-87958-fig3-figsupp1-data1.zip › Figure3 - Figure Supplement 1 - Source Data 1.jfif]

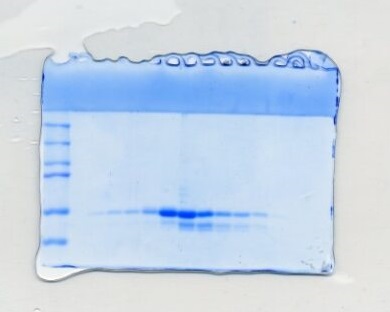

Supplement: Figure 3—figure supplement 2—source data 1. [file elife-87958-fig3-figsupp2-data1.zip › Figure3 - Figure Supplement 2 - Source Data 1.jfif]

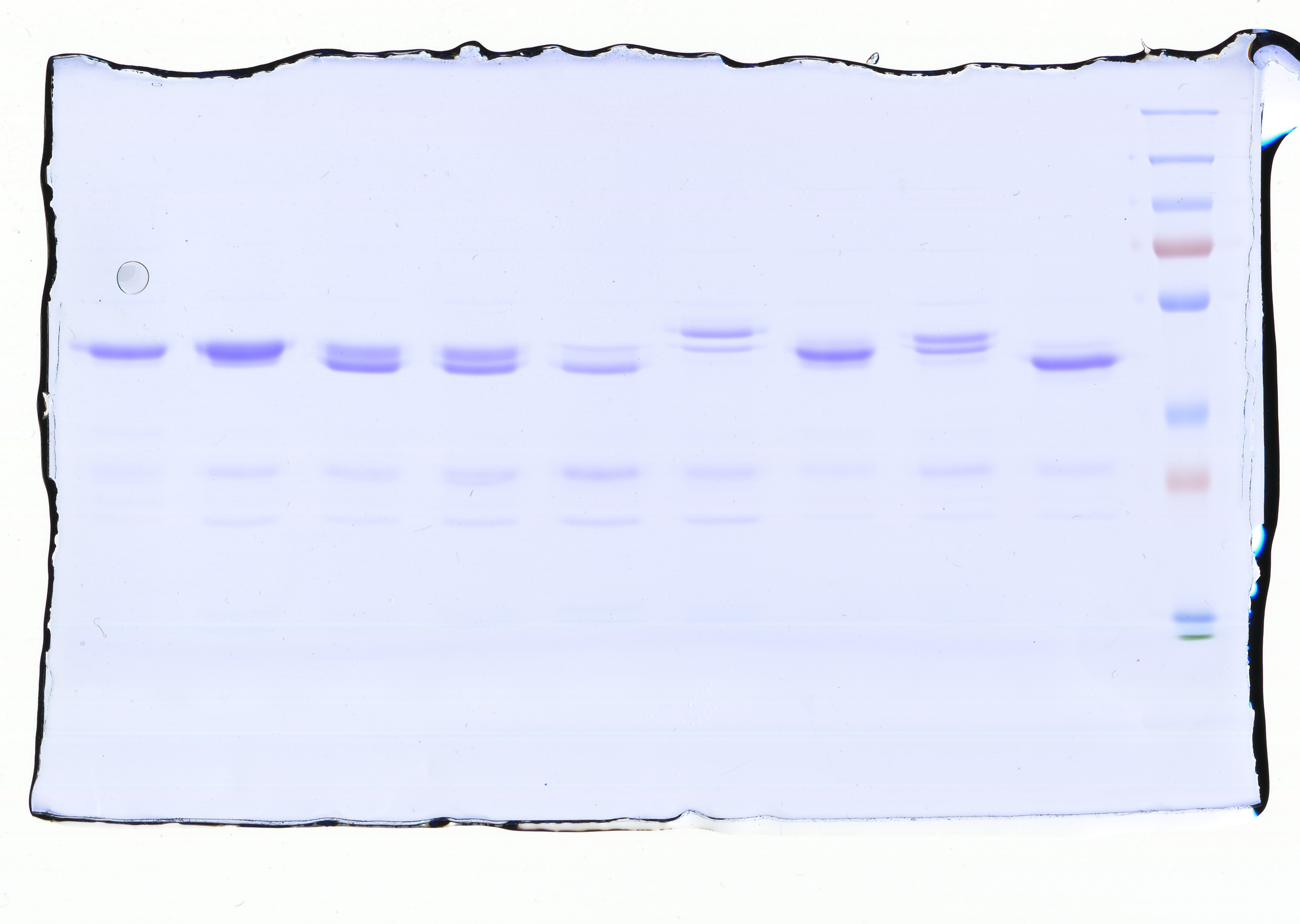

Supplement: Figure 5—figure supplement 1—source data 1. [file elife-87958-fig5-figsupp1-data1.zip › Figure5-figuresupplement1-sourcedata1.jpg]

## Slide 1
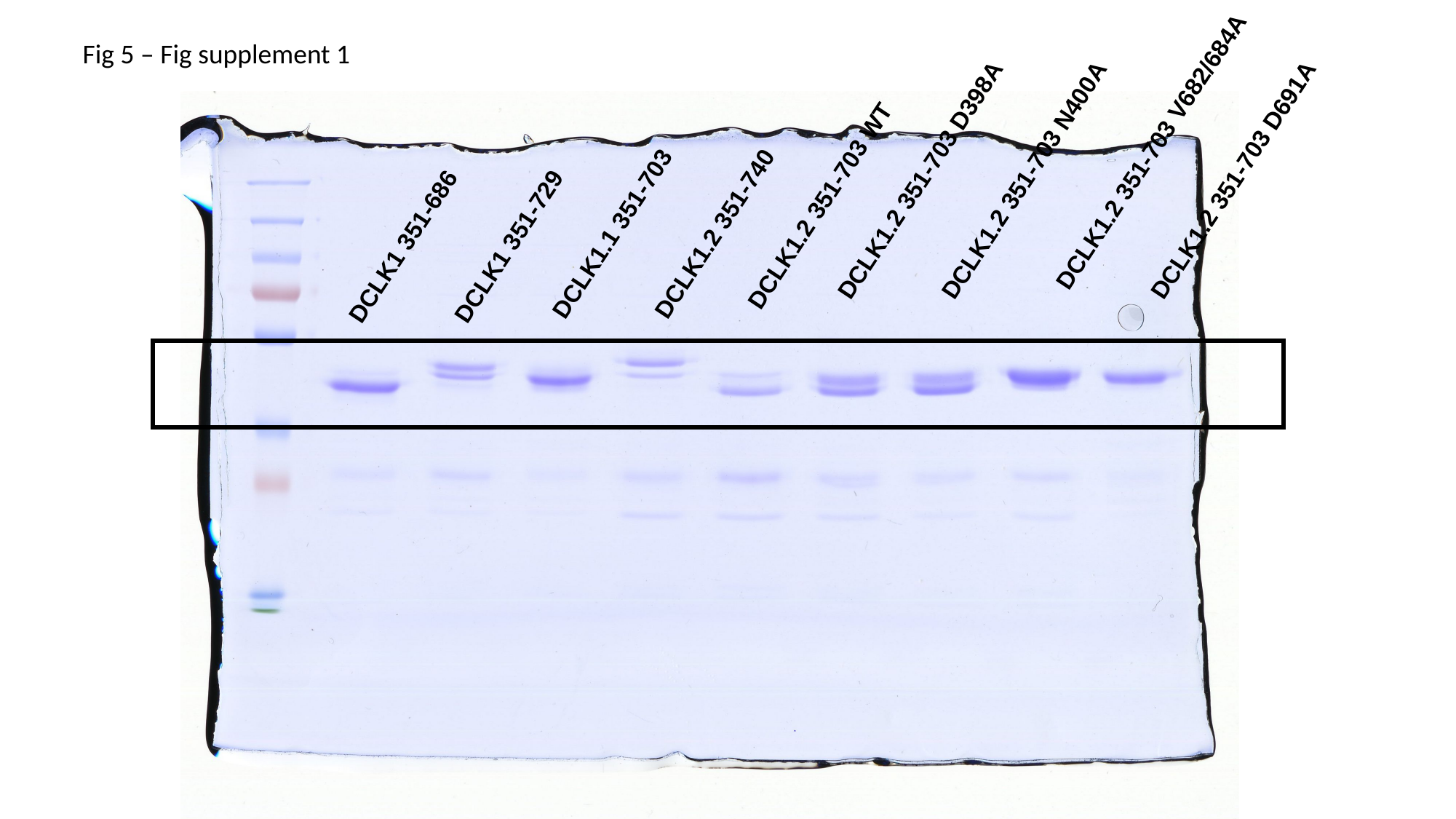

Fig 5 – Fig supplement 1
DCLK1.2 351-703 V682/684A
DCLK1.2 351-703 D398A
DCLK1.2 351-703 N400A
DCLK1.2 351-703 D691A
DCLK1.2 351-703 WT
DCLK1.1 351-703
DCLK1.2 351-740
DCLK1 351-686
DCLK1 351-729

Supplement: Figure 5—figure supplement 1—source data 2. [file elife-87958-fig5-figsupp1-data2.pptx]
